# Supplementary material for: Resolving the full spectrum of human genome variation using Linked-Reads
Source: Genome Res. 2019 Apr;29(4):635–45. doi: 10.1101/gr.234443.118 (PMC6442396; doi:10.1101/gr.234443.118)
Supplement: Supplemental Material [file supp_gr.234443.118_Supplemental_longranger-2.2.zip › longranger-2.2.tar.bz2!/lr2.2-public/lib/stan/cmdstan/stan/doc/api/html/agrad_2rev_2matrix_2multiply_8hpp.html]

Stan: src/stan/agrad/rev/matrix/multiply.hpp File Reference


|  |  |
| --- | --- |
|  | Stan  2.5.0  probability, sampling & optimization |


- Main Page
- Related Pages
- Namespaces
- Classes
- Files

- File List
- File Members

All Classes Namespaces Files Functions Variables Typedefs Enumerations Enumerator Friends Macros Pages

- src
- stan
- agrad
- rev
- matrix

Namespaces |
Functions

multiply.hpp File Reference

`#include <stan/agrad/rev/var.hpp>`  
`#include <stan/agrad/rev/matrix/Eigen_NumTraits.hpp>`  
`#include <stan/agrad/rev/matrix/typedefs.hpp>`  
`#include <stan/math/matrix/Eigen.hpp>`  
`#include <stan/math/matrix/typedefs.hpp>`  
`#include <stan/math/error_handling/matrix/check_multiplicable.hpp>`  
`#include <stan/agrad/rev/matrix/to_var.hpp>`  
`#include <stan/agrad/rev/matrix/dot_product.hpp>`  
`#include <stan/agrad/rev/operators/operator_multiplication.hpp>`  
`#include <boost/utility/enable_if.hpp>`  
`#include <boost/type_traits.hpp>`  
`#include <boost/math/tools/promotion.hpp>`

Go to the source code of this file.

|  |  |
| --- | --- |
| Namespaces | |
|  | stan |
|  | Probability, optimization and sampling library. |
|  | |
|  | stan::agrad |
|  | Function gradients via reverse-mode automatic differentiation. |
|  | |

|  |  |
| --- | --- |
| Functions | |
| template<typename T1 , typename T2 > | |
| boost::enable\_if\_c  < (boost::is\_scalar< T1 >  ::value\|\|boost::is\_same< T1,   var >::value)&&(boost::is\_scalar  < T2 >::value\|\|boost::is\_same  < T2, var >::value), typename   boost::math::tools::promote\_args  < T1, T2 >::type >::type | stan::agrad::multiply (const T1 &v, const T2 &c) |
|  | Return the product of two scalars. More... |
|  | |
| template<typename T1 , typename T2 , int R2, int C2> | |
| Eigen::Matrix< var, R2, C2 > | stan::agrad::multiply (const T1 &c, const Eigen::Matrix< T2, R2, C2 > &m) |
|  | Return the product of scalar and matrix. More... |
|  | |
| template<typename T1 , int R1, int C1, typename T2 > | |
| Eigen::Matrix< var, R1, C1 > | stan::agrad::multiply (const Eigen::Matrix< T1, R1, C1 > &m, const T2 &c) |
|  | Return the product of scalar and matrix. More... |
|  | |
| template<typename T1 , int R1, int C1, typename T2 , int R2, int C2> | |
| boost::enable\_if\_c  < boost::is\_same< T1, var >  ::value\|\|boost::is\_same< T2,   var >::value, Eigen::Matrix  < var, R1, C2 > >::type | stan::agrad::multiply (const Eigen::Matrix< T1, R1, C1 > &m1, const Eigen::Matrix< T2, R2, C2 > &m2) |
|  | Return the product of the specified matrices. More... |
|  | |
| template<typename T1 , int C1, typename T2 , int R2> | |
| boost::enable\_if\_c  < boost::is\_same< T1, var >  ::value\|\|boost::is\_same< T2,   var >::value, var >::type | stan::agrad::multiply (const Eigen::Matrix< T1, 1, C1 > &rv, const Eigen::Matrix< T2, R2, 1 > &v) |
|  | Return the scalar product of the specified row vector and specified column vector. More... |
|  | |


---

[ Stan Home Page ]

*© 2011–2014,
Stan Development Team.*
